# Clostridial Glucosylating Toxins Enter Cells via Clathrin-Mediated Endocytosis

**DOI:** 10.1371/journal.pone.0010673

**Published:** 2010-05-17

**Authors:** Panagiotis Papatheodorou, Constantinos Zamboglou, Selda Genisyuerek, Gregor Guttenberg, Klaus Aktories

**Affiliations:** Institut für Experimentelle und Klinische Pharmakologie und Toxikologie, Albert-Ludwigs-Universität Freiburg, Freiburg, Germany; Universidad Nacional, Costa Rica

## Abstract

*Clostridium difficile* toxin A (TcdA) and toxin B (TcdB), *C. sordellii* lethal toxin (TcsL) and *C. novyi* α-toxin (TcnA) are important pathogenicity factors, which represent the family of the clostridial glucosylating toxins (CGTs). Toxin A and B are associated with antibiotic-associated diarrhea and pseudomembraneous colitis. Lethal toxin is involved in toxic shock syndrome after abortion and α-toxin in gas gangrene development. CGTs enter cells via receptor-mediated endocytosis and require an acidified endosome for translocation of the catalytic domain into the cytosol. Here we studied the endocytic processes that mediate cell internalization of the CGTs. Intoxication of cells was monitored by analyzing cell morphology, status of Rac glucosylation in cell lysates and transepithelial resistance of cell monolayers. We found that the intoxication of cultured cells by CGTs was strongly delayed when cells were preincubated with dynasore, a cell-permeable inhibitor of dynamin, or chlorpromazine, an inhibitor of the clathrin-dependent endocytic pathway. Additional evidence about the role of clathrin in the uptake of the prototypical CGT family member toxin B was achieved by expression of a dominant-negative inhibitor of the clathrin-mediated endocytosis (Eps15 DN) or by siRNA against the clathrin heavy chain. Accordingly, cells that expressed dominant-negative caveolin-1 were not protected from toxin B-induced cell rounding. In addition, lipid rafts impairment by exogenous depletion of sphingomyelin did not decelerate intoxication of HeLa cells by CGTs. Taken together, our data indicate that the endocytic uptake of the CGTs involves a dynamin-dependent process that is mainly governed by clathrin.

## Introduction

*Clostridium difficile* toxin A (TcdA) and toxin B (TcdB), *Clostridium sordellii* lethal toxin (TcsL) and *Clostridium novyi* α-toxin (TcnA) are important pathogenicity factors of the family of clostridial glucosylating toxins (CGTs). Toxin A and B are the main cause of antibiotic-associated diarrhea and pseudomembraneous colitis [1], lethal toxin is implicated in toxic shock syndrome after medical-induced abortion [2] and α-toxin causes gas gangrene syndrom [3],[4].

CGTs consist of at least four domains [5]. At the N-terminus, the glycosyltransferase domain is located [6], which modifies low molecular mass GTP-binding proteins of the Rho and/or Ras family by mono-O-glucosylation [7],[8] or mono-O-GlcNAcylation (α-toxin) [9]. An adjacent cysteine protease domain releases the glucosyltransferase into the cytosol by autoproteolytic cleavage [10]. The middle portion of the toxins is considered to mediate membrane insertion during the translocation process and is probably responsible for pore formation in membranes. Finally, target cell binding is mainly mediated by the C-terminal domain, which is characterized by repetitive oligopeptides (CROPs) [11],[12].

CGTs enter cells by receptor-mediated endocytosis and require an acidic endosomal compartment for complete translocation of the enzyme moiety into the cytosol [13],[14],[15]. To date, only for toxin A binding sites at the cell surface have been described, namely carbohydrates, including the trisaccharide Galα1-3Galβ1-4GlcNac or protein receptors like sucrase-isomaltase and the glycoprotein gp96 [16],[17],[18]. Far less is known about the endocytic mechanisms underlying the internalization of the clostridial glucosylating toxins.

Endocytosis of molecules is either mediated by clathrin-coated pits or by clathrin-independent mechanisms, subdivided into Rac-, RhoA-, Cdc42-, Arf6- or caveolar-regulated uptake pathways [19],[20]. So far, bacterial toxins have evolved into utilizing all known cell entry points [21].

Here we studied the endocytic processes that mediate cell internalization of the CGTs, using pharmacological substances and genetical approaches that impair certain endocytic pathways. We show that the route to intracellular compartments for this toxin family is mediated by a dynamin-dependent process governed by clathrin. Our study additionally excludes the involvement of lipid rafts during clathrin-dependent uptake of the CGTs.

## Results

### Uptake of CGTs into cells depends on dynamin

**Figure 1:**
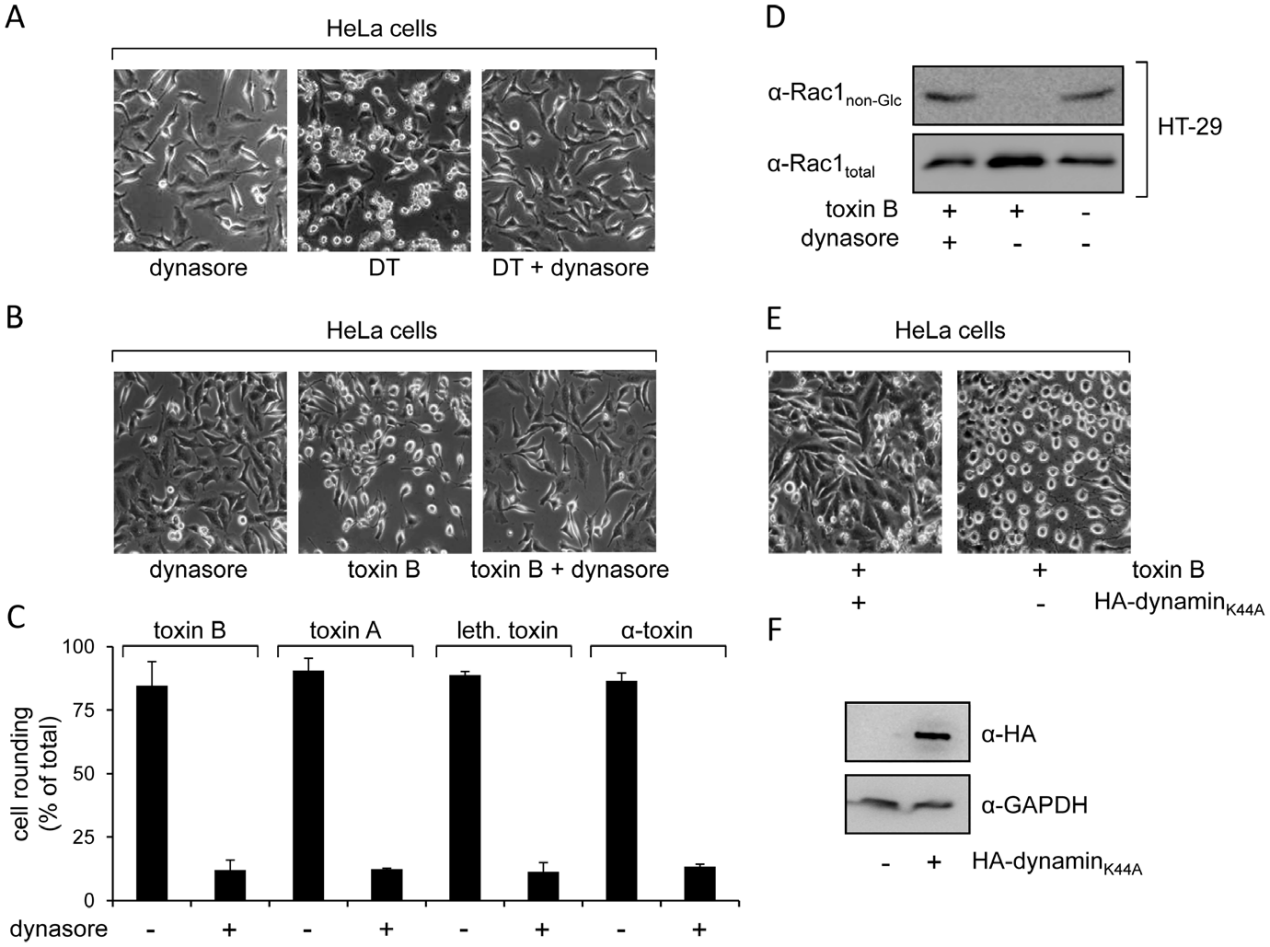
Inhibition of dynamin with dynasore and a dominant-negative mutant. Dynasore-pretreated and non-pretreated HeLa cells were incubated with (A) 5 nM diphtheria toxin for 180 min or (B) 4 pM toxin B for 75 min, prior to microscopical analysis of the cell morphology. (C) HeLa cells preincubated with dynasore or with solvent only were treated with 4 pM toxin B for 75 min, 1.5 nM toxin A for 105 min, 4 nM lethal toxin for 75 min or 1.5 nM α-toxin for 60 min. The percentage of rounded cells was quantified and data are given +/− SD (n = 3) and from a minimum of 200 cells in total. (D) Dynasore-pretreated HT-29 cells and untreated cells were intoxicated with 40 pM toxin. After onset of cell rounding (150 min), glucosylation status of Rac1 in cell lysates was analyzed with antibodies recognizing either only unmodified Rac1 (α-Rac1_non-Glc_) or all Rac1 (α-Rac1_total_). (E) HeLa cells expressing dominant-negative dynamin (HA-dynamin_K44A_) for 24 h or mock-transfected cells were intoxicated with 4 pM toxin B for 75 min and cell morphology analyzed microscopically. (F) Selective expression of HA-dynamin_K44A_ in plasmid-transfected HeLa cells, but not in mock-transfected cells, was detected in cell lysates with an anti-HA antibody. Antibody detection of the housekeeping protein glyceraldehyde 3-phosphate dehydrogenase (α-GAPDH) served as a control for equal loading of lysate samples.

The GTPase dynamin is involved in the pinch-off of endocytic vesicles from the plasma membrane. Therefore, dynamin-dependency confines the endocytic uptake mechanism for a given molecule to clathrin-, caveolae- and RhoA-mediated pathways [20]. To test whether internalization of CGTs requires dynamin, dynasore, a potent cell-permeable inhibitor of dynamin [22],[23], was preincubated with HeLa cells, prior to addition of toxins. Diphtheria toxin that is endocytosed via clathrin-coated pits in a dynamin-dependent manner [24] was used as a positive control. Accordingly, the cytopathic effect of diphtheria toxin on HeLa cells was strongly inhibited when cells were pretreated with dynasore (Fig. 1A). Intoxication of HeLa cells with CGTs leads to cell rounding, due to the inactivation of Rho proteins, which regulate the actin cytoskeleton as well as microtubule-based structures [25]. Dynasore conferred resistance towards cell rounding in HeLa cells incubated with the prototypic member of the CGT family, *C. difficile* toxin B (Fig. 1B). Interestingly, cell rounding induced by toxin B, toxin A, lethal toxin and α-toxin was equally reduced to ∼10% in dynasore-pretreated cells, when compared with non-pretreated cells (85–90% cell rounding) (Fig. 1C). The importance of dynamin in the uptake of CGTs was also tested with toxin B in the human colon adenocarcinoma cell line HT-29 (Cell Line Services, Eppelheim, Germany). Toxin B-induced intoxication was monitored by analysis of the glucosylation status of Rac1 in HT-29 cell lysates, using a specific anti-Rac1 antibody that recognizes only non-glucosylated Rac1. As expected, glucosylation of Rac1 in HT-29 cells treated with toxin B, was prevented by preincubation of cells with dynasore (Fig. 1D). Additional evidence about the importance of dynamin in the uptake of toxin B was obtained by intoxication of HeLa cells expressing dominant-negative dynamin (HA-dynamin_K44A_). Toxin B-induced cell rounding was strongly inhibited in HA-dynamin_K44A_–transfected HeLa cells when compared to mock-transfected cells (Fig. 1E). Expression of HA-dynamin_K44A_ was detected in HeLa cell lysates by the use of an anti-HA antibody (Fig. 1F). Taken together, data obtained in HeLa and/or HT-29 cells using dynasore or dominant-negative dynamin, strongly indicate the involvement of a dynamin-dependent endocytic pathway in the cellular uptake of CGTs.

### Pharmacological inhibition of clathrin-coated pits formation prevents uptake of CGTs

**Figure 2:**
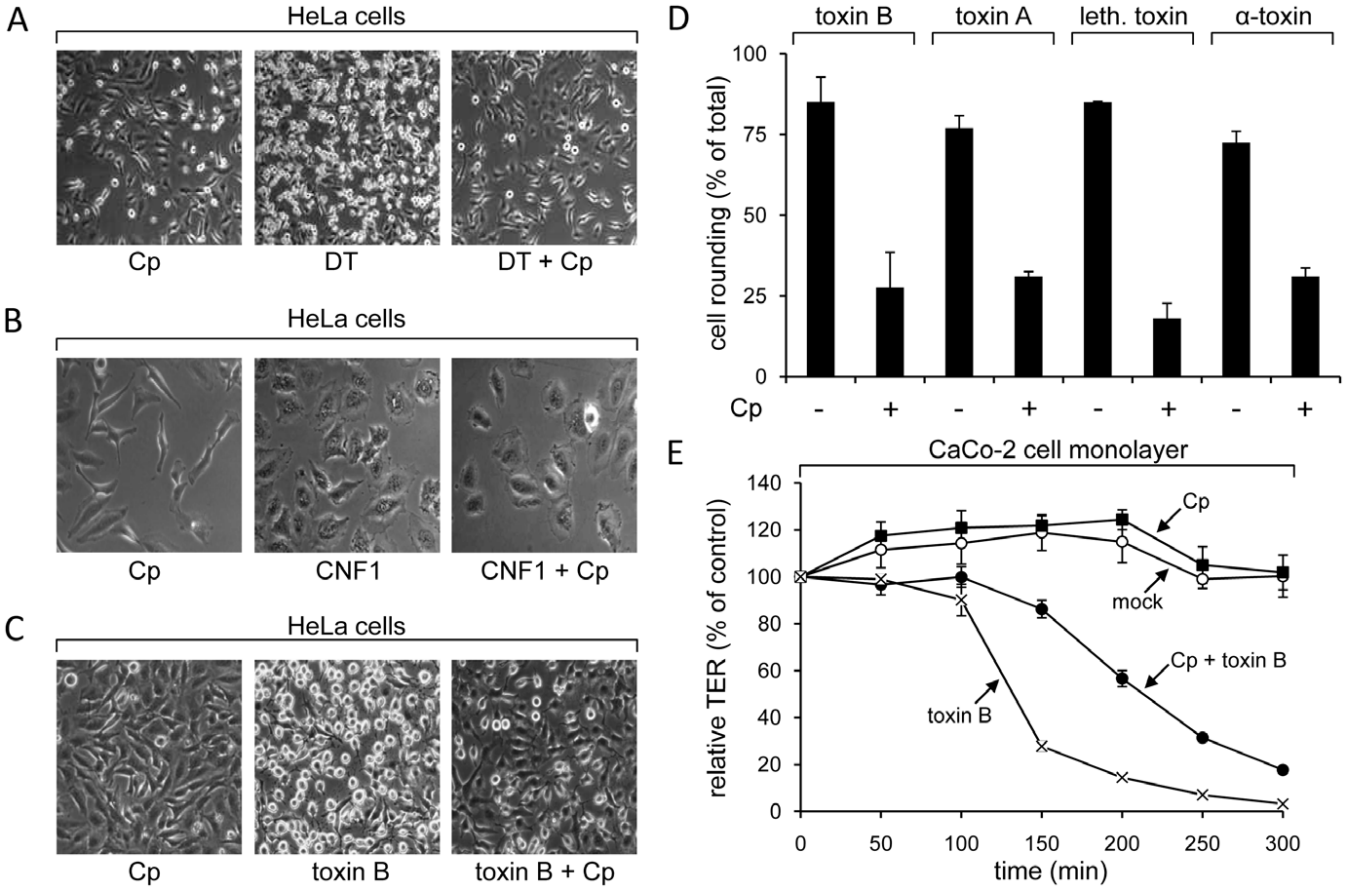
Pharmacological inhibition of clathrin assembly with chlorpromazine. HeLa cells were preincubated with chlorpromazine (Cp) or left untreated, prior to addition of (A) 5 nM diphtheria toxin (DT) and incubation for 180 min, (B) 3.5 nM CNF1 and incubation for 150 min or (C) 4 pM toxin B and incubation for 75 min. (D) The percentage of cell rounding in chlorpromazine-pretreated or non-pretreated HeLa cells after intoxication with 4 pM toxin B for 75 min, 1.5 nM toxin A for 105 min, 4 nM lethal toxin for 75 min or 1.5 nM α-toxin for 60 min was quantified and data are given +/− SD (n = 3) and from a minimum of 200 cells in total. (E) Human intestinal epithelial cells (CaCo-2) were grown to confluency on filters and were preincubated with chlorpromazine (Cp) or left untreated. A subset of cells was intoxicated with 40 pM toxin B and transepithelial electrical resistance (TER) was measured at indicated time points, where starting resistance was set to 100% and TER values are calculated as relative TER in % from starting resistance (+/− SD, n = 3).

Clathrin is a coat protein of specific endocytic vesicles, mediating the internalization of a wide range of transmembrane receptors and their ligands [26]. Since clathrin-dependent endocytosis requires dynamin, this endocytic pathway could be relevant for the uptake of CGTs that are sensitive to dynamin inhibition. To test the importance of clathrin in the endocytosis of CGTs, we used chlorpromazine, a drug that prevents formation of clathrin-coated pits at the plasma membrane [27]. HeLa cells preincubated with chlorpromazine showed strong resistance against cytopathic effects induced by the diphtheria toxin, which served again as a positive control (Fig. 2A). This was not the case for CNF1, a Rho-deamidating toxin from pathogenic *Escherichia coli* leading to cell flatenning and polynucleation [28]. CNF1 enters cells by clathrin-independent mechanisms [29] and thereby confirms the selective inhibition of clathrin-dependent pathways by this drug (Fig. 2B). Importantly, chlorpromazine-pretreated HeLa cells were less sensitive to cell rounding induced by toxin B, toxin A, lethal toxin and α-toxin (∼20–30% cell rounding), when compared with cells not pretreated with this drug (∼75–85% cell rounding) (Fig. 2C and 2D). The CGTs-inhibiting effect of chlorpromazine in HeLa cells was paradigmatically further verified with toxin B in the human epithelial colorectal adenocarcinoma cell line Caco-2 (Cell Line Services, Eppelheim, Germany), by measuring changes in the transepithelial resistance of confluent monolayers. Toxin B-induced drop of the transepithelial resistance in the Caco-2 cell monolayer was attenuated, when cells were preincubated with chlorpromazine (Fig. 2E). Taken together, the pharmacological tool chlorpromazine applied as an inhibitor of the formation of clathrin-coated pits, indicated an involvement of this endocytic compartment in the cellular internalization of CGTs.

### RNAi-mediated gene silencing of clathrin heavy chain impedes toxin B intoxication

**Figure 3:**
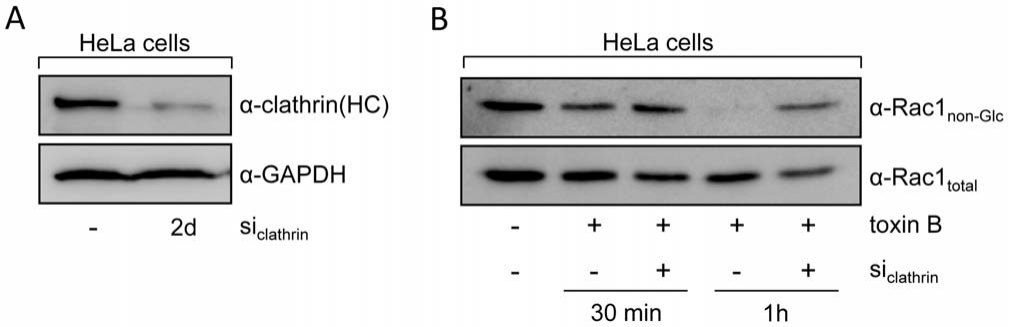
RNAi-mediated gene silencing of the clathrin heavy chain. HeLa cells were transfected with siRNA against the clathrin heavy chain (si_clathrin_). (A) Two days after transfection, cell lysates from si_clathrin_-transfected cells and mock-transfected cells were analyzed for clathrin heavy chain expression using a specific antibody (α-clathrin(HC)). Antibody detection of the housekeeping protein glyceraldehyde 3-phosphate dehydrogenase (α-GAPDH) served as a control for equal loading of lysate samples. (B) After addition of toxin B (4 pM), Rac1 glucosylation status was compared in lysates from si_clathrin_- and mock-transfected HeLa cells at indicated time points with antibodies recognizing either only unmodified Rac1 (α-Rac1_non-Glc_) or all Rac1 (α-Rac1_total_).

The involvement of clathrin in the uptake of CGTs was further analyzed by transfection of siRNA directed against the clathrin heavy chain to impair the formation of clathrin-coated pits. Two days after transfection of HeLa cells with siRNA, glucosylation of Rac1 upon addition of toxin B and incubation for 30 and 60 min was compared between siRNA- and mock-transfected cells. Reduced protein levels of clathrin heavy chain in lysates of siRNA-treated cells were confirmed by the use of an anti-clathrin heavy chain antibody (Fig. 3A). As expected, cells transfected with siRNA against the clathrin heavy chain retained higher amounts of non-glucosylated Rac after 30 and 60 min of toxin-treatment, when compared with mock-transfected cells (Fig. 3B). This result substantiates the importance of clathrin-mediated processes in the endocytic uptake of CGTs.

### Clathrin- rather than caveolae-mediated endocytic mechanisms are implicated in the cellular internalization of toxin B

**Figure 4:**
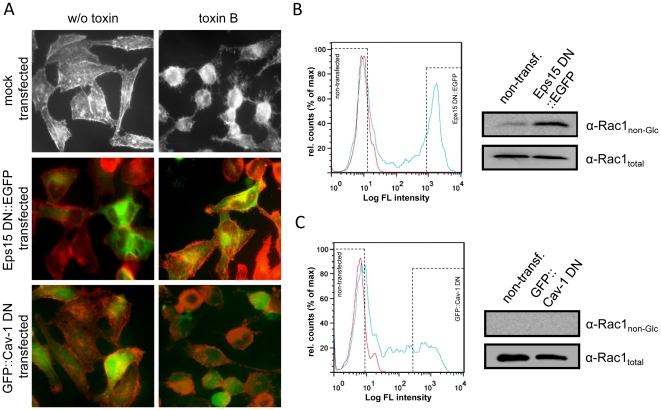
Expression of dominant-negative Eps15 and Cav-1. (A) HeLa cells, transfected with plasmids, encoding dominant-negative forms of Eps15 or Cav-1 fused to a GFP moiety, were eventually intoxicated for 75 min with 4 pM toxin B or left untreated. Toxin B-induced alterations in cell morphology were analyzed in Eps15- (Eps15 DN::EGFP), Cav-1- (GFP::Cav-1 DN) and mock-transfected cells after actin staining with FITC-phalloidin (red and grey signals) and by applying fluorescence microscopy. Green signals derived from cells expressing GFP-tagged, dominant-negative Eps15 or Cav-1, respectively. (B) HeLa cells were transfected with a plasmid encoding Eps15 DN::EGFP, intoxicated with 4 pM toxin B for 30 min and subsequently subjected to fluorescence-assisted cell sorting. GFP excitation of the Eps15 DN::EGFP-transfected cells resulted in two cell populations (left panel, blue curve, bordered with dashed line). One population represents non-transfected cells, with overlapping background fluorescence as obtained in mock-transfected cells (left panel, red curve). The other population represents Eps15 DN::EGFP-expressing cells. Equal number of cells from both cell populations (non-transf. and Eps15 DN::EGFP) where subjected to cell lysis and analysis of the Rac1 glucosylation status with antibodies recognizing either only unmodified Rac1 (α-Rac1_non-Glc_) or all Rac1 (α-Rac1_total_). (C) Procedure was performed essentially as described in (B), but with HeLa cells transfected with a plasmid encoding GFP::Cav-1.

To discriminate between the two major endocytic pathways that include dynamin (clathrin- and caveolae-pathway), plasmids encoding a dominant-negative variant of Cav-1 (Cav-1 DN, caveolae pathway) or Eps15 (Eps15 DN, clathrin pathway) were transfected in HeLa cells. Both proteins are fused to a GFP moiety and enable for the direct observation of the transfected cells by fluorescence microscopy. Importantly, addition of toxin B led to cell rounding predominantly in non-transfected and Cav-1 DN-transfected cells (81.9%, SD +/−10.4%, of total cells and 82.1%, SD +/−12.9%, of total cells, respectively), and to much lesser extent in Eps15 DN-transfected cells (26.2%, SD +/−14.8%, of total cells) (Fig. 4A). The selective protection of cells expressing dominant-negative Eps15 against toxin B was confirmed by analyzing the Rac1 glucosylation status of Cav-1 DN- or Eps15 DN-transfected HeLa cells. For this purpose, transfected cells (expressing a GFP moiety fused to Cav-1 DN or Eps15 DN) were separated from non-transfected cells by applying fluorescence-assisted cell sorting, subsequently after preincubation with toxin B. Strikingly, Rac1 glucosylation was prevented exclusively in cells expressing dominant-negative Eps15 (Fig. 4B), but not in cells expressing dominant-negative Cav-1 (Fig. 4C) or non-transfected cells (Fig. 4B and 4C). Conclusively, this result finally proves the predominant role of clathrin- rather than caveolae-mediated processes in the endocytic uptake of CGTs.

### Lipid rafts are not implicated in the endocytic uptake of CGTs

**Figure 5:**
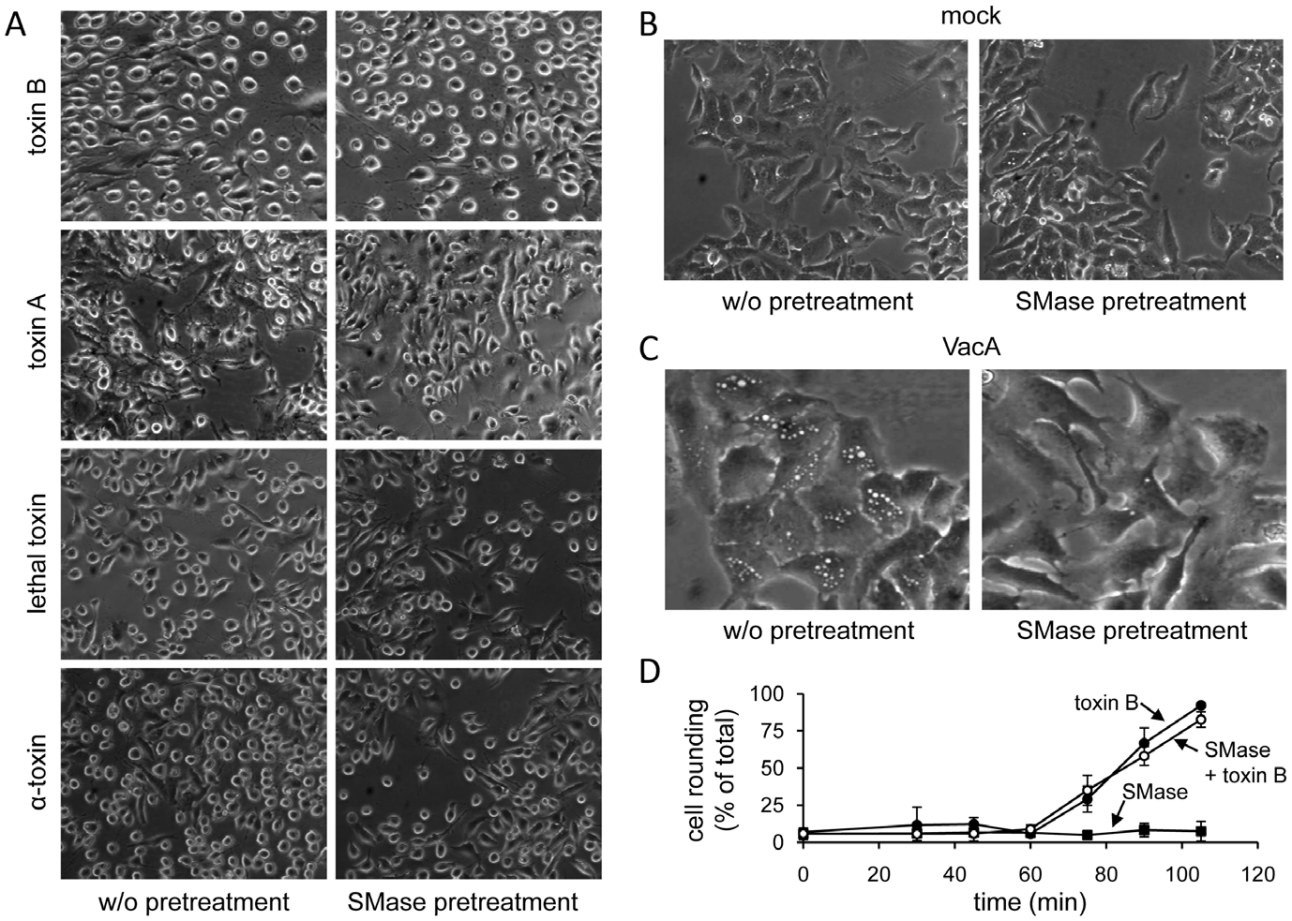
Disintegration of lipid rafts by exogenous sphingomyelin depletion with SMase. HeLa cells were pretreated with SMase or left untreated prior to intoxication with (A) 4 pM toxin B for 75 min, 5 nM toxin A for 300 min, 5 nM lethal toxin for 180 min, 5 nM α-toxin for 120 min, (B) mock incubation for 300 min, or (C) 50 nM VacA toxin for 300 min. Images were obtained by microscopy, upon onset of intoxication characteristics. (D) SMase-preincubated or non-preincubated HeLa cells were intoxicated with 4 pM toxin B or left untreated. The percentage of cell rounding was quantified from three independent experiments at indicated time points (data are given +/− SD).

Caveolae-mediated endocytic processes are exclusively taking place at microdomains of the plasma membrane denoted as lipid rafts [30]. But there is growing evidence for the implication of lipid rafts also in clathrin-dependent uptake mechanisms [31],[32],[33]. Therefore, we wanted to study the role of lipid rafts in the uptake of the CGTs. Sphingomyelin is a prominent component of lipid rafts and can be exogenously depleted from cell membranes by the addition of the enzyme sphingomyelinase (SMase) from *Bacillus cereus* [34]. In HeLa cells pretreated with SMase, no significant delay in cell rounding was observed upon addition of toxin B, toxin A, lethal toxin or α-toxin, when compared with non-pretreated cells (Fig. 5A). Control cells that were incubated with SMase alone did not show morphological alterations when compared with untreated cells (Fig. 5B). The exogenous depletion of sphingomyelin in HeLa cells was evaluated by using the vacuolating cytotoxin A (VacA) from *Helicobacter pylori* as a marker for sphingomyelin-/lipid rafts-dependent entry into host cells [35],[36]. As expected, vacuolization in HeLa cells upon addition of VacA was strongly reduced in sphingomyelin-depleted cells, when compared with control cells under equal conditions (85% reduction) (Fig. 5C). The lack of protection of HeLa cells from toxin B-induced cell rounding by SMase pretreatment was further confirmed in a time-dependent manner (Fig. 5D). In conclusion, lipid rafts seem not to be crucially involved in processes leading to the endocytic uptake of CGTs.

## Discussion

Toxins of the family of clostridial glucosylating toxins are considered to enter cells by receptor-mediated endocytosis [37]. In contrast to toxins which finally reach their cytosolic substrates by a retrograde transport via the Golgi apparatus and the endoplasmatic reticulum (e.g. cholera toxin) [38], CGTs seem to require acidified early endosomes for the delivery of their enzymatic part into the cytosol [14],[15]. So far, the mechanisms underlying internalization of CGTs into endosomal compartments were not investigated in detail. Kushnaryov *et al.* provided evidence for endocytosis of *C. difficile* toxin A via coated pits by visualizing colloidal gold labeled toxin A in CHO cells by electron microscopy [37]. However, it remained unclear, whether these coated pits are functionally relevant for intoxication. We studied the internalization of CGTs in detail by using *C. difficile* toxin B as a model protein. At least for toxin B, three independent lines of evidence indicate the involvement of clathrin in its uptake into cells. First, pharmacological inhibition of clathrin-coated pits formation using chlorpromazine strongly reduced cytopathic effects of toxin B in HeLa and Caco-2 cells. Second, siRNA targeted against the clathrin heavy chain strongly reduced Rac1 glucosylation in HeLa cells by toxin B. Third, ectopic expression of dominant-negative Eps15 (participates in assembly of clathrin-coated pits) retained toxin B-induced cell rounding and Rac glucosylation in HeLa cells. CGTs are considered to use different receptor structures on the cell surface for binding and internalization, leading to endocytic uptake mechanisms that are possibly unique for each member of the CGT family. Since the pharmacological inhibition of the clathrin pathway with chlorpromazine also blocked intoxication of HeLa cells with toxin A, lethal toxin and α-toxin, we conclude that members of the CGT family at least share a common mechanism for cell entry.

Pinch-off of clathrin-coated vesicles from the plasma membrane requires the action of the GTPase dynamin. In addition, dynamin confers the constriction of caveolar vesicles from the plasma membrane [39]. Consistent with the finding that CGT uptake is mediated by clathrin, inhibition of dynamin activity with dynasore prevented CGT-induced cell rounding in HeLa cells and toxin B-induced Rac1 glucosylation in HT-29 cells. Inhibition of *C. difficile* toxin B intoxication of HeLa cells by expressing dominant-negative dynamin confirms the participation of this GTPase in the toxin uptake. The involvement of caveolin in the uptake of *C. difficile* toxin B could be excluded, since ectopic expression of dominant-negative Cav-1 in HeLa cells did not prevent toxin B-induced cell rounding and Rac1 glucosylation. The fact that cells, which do not express detectable levels of caveolin, e.g. HepG2 [40] or Caco-2 cells [41], can readily be intoxicated by toxin B (data not shown), argues also against a caveolae-mediated internalization of this toxin. Moreover, caveosomes are neutral and do not communicate with other acidic compartments [42]. Internalization in such endocytic vesicles is therefore unlikely, due to the fact that transport of the enzymatic part of *C. difficile* toxin B into the cytosol requires low pH [14],[15]. Even more, caveolae exclusively arise from membrane microdomains denoted as lipid rafts [30]. Since toxin B, toxin A, lethal toxin and α-toxin were not negatively influenced after disruption of lipid rafts by exogenous sphingomyelin depletion, this result provides additional evidence that cell entry of all CGTs does not occur through caveolae.

Our experimental data does not directly exclude a minor participation of other clathrin-independent endocytic pathways, such as the RhoA- or Cdc42-regulated endocytosis, in the uptake of CGTs. Even though both GTPases can be modified and inactivated by the clostridial glucosylating toxins, with the exception of the lethal toxin that does not modify RhoA, their requirement in initial stages of the intoxication process is still conceivable. However, Cheng and coworkers reported *in vivo* membrane binding of both GTPases to depend on the presence of sphingomyelin in the plasma membrane [43]. They showed that upon incubation of cells with SMase to hydrolyze sphingomyelin at the cell surface, RhoA- and Cdc42-dependent endocytosis of cargo proteins was blocked. Based on these findings and the fact that CGTs intoxication occurs also in sphingomyelin-depleted cells, it is unlikely that RhoA- or Cdc42-mediated endocytic pathways participate in cell entry of those toxins.

Our study provides an evidence for the clathrin-dependent endocytic uptake of clostridial glucosylating toxins. The uptake process might serve as a target for therapeutical intervention in the pathogenesis of clostridial infections. Based on our findings, it is also expected that CGTs may be used as novel probes for studying cell entry of other cargo proteins with unknown uptake mechanism.

## Materials and Methods

### Toxins and plasmids used in this study

Toxin B and lethal toxin used in this study were recombinantly purified from *Bacillus megaterium* with protocols as published by others previously for *Clostridium difficile* toxin A and toxin B [44]. Native toxin A and α-toxin were used in this study, purified from *C. difficile* (strain VPI 10463) and *C. novyi* (strain ATCC 19402), respectively, according to protocols described elsewhere [45],[46]. Toxin A was additionally purified by thyroglobuline affinity chromatography [47]. Recombinant *E. coli* CNF1 protein was allocated by G. Schmidt (University of Freiburg, Germany). Diphtheria toxin was ordered from Calbiochem (Darmstadt, Germany). Native VacA toxin (allelic type s1a/m1) from concentrated *Helicobacter pylori* (strain Hp402022A) culture supernatans, was kindly provided by M. Kist (National Reference Center for *Helicobacter pylori*, University of Freiburg, Germany). VacA was acid-activated and re-neutralized prior to usage in intoxication assays. Plasmids encoding dominant-negative Eps15, Cav-1 or dynamin were generous gifts of A. Benmerah (INSERM, Paris, France), J. Eggermont (University of Leuven, Belgium) and C. van Koppen (University of Essen, Germany), respectively.

### Antibodies

Proteins were separated by SDS-PAGE and transferred onto a polyvinylidene difluoride membrane (PVDF) for antigen detection using specific antibodies. Clathrin heavy chain was detected using a purified mouse anti-Clathrin Heavy Chain antibody (#610499, BD Transduction Laboratories). Glucosylated and non-glucosylated Rac1 was detected with an anti-Rac1 monoclonal antibody clone 23A8 (#05-389, Millipore); anti-Rac1 monoclonal antibody clone 102 (#610650, BD Transduction Laboratories) was used for specific detection of non-glucosylated Rac1 only. GAPDH was detected with a monoclonal anti-GAPDH antibody clone 6C5 (#MAB374, Millipore). HA-tagged proteins were detected with a rabbit polyclonal anti-HA antibody (#H6908, Sigma-Aldrich).

### Cell cultivation and preparation of cell lysates

HeLa and Caco-2 cells were grown at 37°C with 5% CO_2_ in Dulbecco's modified Eagle's medium (DMEM) (12 mM L-glutamine) supplemented with 10% fetal calf serum (FCS), 1% non-essential amino acids (NEA), penicillin (4 mM) and streptomycin (4 mM), and for Caco-2 cells only, 1% sodium pyruvate (1 mM). HT-29 cells were grown at same conditions in McCoy's medium supplemented with 10% FCS and 1% penicillin (4 mM) and streptomycin (4 mM). Cell lysates of toxin-treated cells were prepared by washing cells twice in phosphate-buffered saline (PBS) and lysis on ice using a buffer containing 50 mM Tris, 100 mM NaCl, 2 mM MgCl_2_, 10% w/v glycerol, 1% Igepal CA-630, 1% SDS and 0.1 mM PMSF, pH 7.4.

### Inhibitor assays for investigation of toxin uptake

Prior to addition of toxins, cells were preincubated for 45 min at 37°C in medium lacking FCS and with substances specifically inhibiting endocytic pathways. HeLa cells were preincubated with 80 µM dynasore or 12.5 µM chlorpromazine; HT-29 cells with 80 µM dynasore or 30 µM chlorpromazine and Caco-2 cells with 30 µM chlorpromazine. Exogenous sphingomyelin depletion in HeLa cells was achieved by direct addition of 100 mU/ml sphingomyelinase from *Bacillus cereus* in culture medium and incubation for 1 h at 37°C. All compounds were purchased from Sigma-Aldrich (Deisenhofen, Germany).

### Microscopy

Mophological changes of intoxicated cells were directly analyzed in wells using an inverted microscope (Axiovert 25, Carl Zeiss Jena GmbH, Germany). Fluorescence signals of cells were analyzed using a fluorescence microscope (Axiophot, Carl Zeiss Jena GmbH, Germany) with a Neofluar 40 object lens and filter set 43H for phalloidin/TRITC or 65 HE for GFP. Images were obtained with an inbuilt digital camera (AxioCam HRc or HRm) and contrast was adjusted manually using AxioVision software (version 3.1.2.1).

### FITC-phalloidin staining of F-actin in cultured cells

Fluorescein isothiocyanate (FITC) conjugated phalloidin was used to label F-actin in HeLa cells. Briefly, cells were grown on cover slips and fixed with 4% (v/v) paraformaldehyde for 15 min prior to permeabilization with 0.1% Triton X-100 for 1 min. Cells were then incubated with 1% (v/v) TRITC-conjugated phalloidin (Invitrogen, Karlsruhe, Germany) for 45 min in the dark. Fixed samples were analyzed with fluorescence microscopy.

### Transfection studies for investigation of toxin uptake

Clathrin heavy chain targeting siRNA, according to HC oligo I from reference [48], was obtained from Dharmacon (Lafayette, USA) and applied for transfection in HeLa cells by using Lipofectamine 2000 (Invitrogen) following the manufacturer's recommendations. The same transfection procedure was used for transfection of a plasmid encoding a dominant-negative dynamin mutant (pRK5/HA-dynamin-1aa_K44A_; [49]) in HeLa cells. Plasmids encoding a dominant-negative form of Eps15 (pEGFP-C2/Eps15DN; [50]) or Cav-1 (pCINeo/Ires-GFP/cav-1DN; [51]) were transfected in HeLa cells either by using Lipofectamine (for subsequent microscopic analysis) or by addition of a mixture of Opti-MEM® I Reduced-Serum Medium (Invitrogen, Karlsruhe, Germany), including 0.005% w/v polyethylenimine and 0.2 µg/ml plasmid DNA, to DMEM medium containing 10% FCS and 1% NEA (for subsequent fluorescence-assisted cell sorting). Following an incubation of 5 h at 37°C, cells were washed with DMEM and further incubated at standard conditions. The effects of dominant-negative Eps15 or Cav-1 expression and siRNA-mediated clathrin gene silencing were assayed one and two days after transfection, respectively.

### Fluorescence-activated cell sorting (FACS)

HeLa cells transfected with a plasmid encoding a GFP-tagged, dominant-negative form of Eps15 or Cav-1 were separated from non-transfected cells by using a MoFlo high speed cell sorter (Dako, Hamburg, Germany). Prior to cell sorting, mock-transfected cells were used to define the parameters for restrictive sorting of single and living cells. An argon ion laser, emitting light at 488 nm (blue), was used for excitation of GFP signals in transfected cells. Data were analyzed with the flow cytometry analysis program FlowJo.

### TER measurement

For analysis of transepithelial resistence (TER) of Caco-2 cell monolayers, cells were grown on filters (Millicell; Millipore) to confluency. TER was measured at indicated time points after toxin-treatment by use of a volt-ohm meter (World Precision Instruments, USA) equipped with a chamber for filter insertion. Only filters with cell monolayers showing an initial resistance of at least 200 Ω per cm^2^ were used.
